# Visualization of Venous Compliance of Superficial Veins Using Non-Contact Plethysmography Based on Digital Red-Green-Blue Images

**DOI:** 10.3390/s16121996

**Published:** 2016-11-25

**Authors:** Kazuya Nakano, Yuta Aoki, Ryota Satoh, Hiroyuki Suzuki, Izumi Nishidate

**Affiliations:** 1 Faculty of Science, Division 2, Tokyo University of Science, 1-3, Kagurazaka, Shinjuku-ku, Tokyo 162-8601, Japan; 2 Graduate School of Bio-Application and Systems Engineering, Tokyo University of Agriculture and Technology, 2-24-16, Naka-cho, Koganei, Tokyo 184-8588, Japan; 50015401127@st.tuat.ac.jp (Y.A.); ryoutasato7assuncao@gmail.com (R.S.); inishi@cc.tuat.ac.jp (I.N.); 3 Laboratory for Future Interdisciplinary Research of Science and Technology, Institute of Innovative Research, Tokyo Institute of Technology, 4259, Nagatsuta-cho, Midori-ku, Yokohama, Kanagawa 226-8503, Japan; hiroyuki@isl.titech.ac.jp

**Keywords:** venous compliance, non-contact measurement, venous thromboembolism

## Abstract

We propose the visualization of venous compliance (VC) using a digital red-green-blue (RGB) camera. The new imaging method, which transforms RGB values into VC, combines VC evaluation with blood concentration estimation from the RGB values of each pixel. We evaluate a non-contact plethysmography (NCPG) system for VC based on comparisons with conventional strain gauge plethysmography (SPG). We conduct in vivo measurements using both systems and investigate their differences by evaluating the VC. The results show that the two methods measure different blood vessels and that errors caused by interstitial fluid accumulation are negligible for the NCPG system, whereas SPG is influenced by such errors. Additionally, we investigate the relationship between VC and physical activity using NCPG.

## 1. Introduction

The daily monitoring of vital signs, such as blood pressure and heart rate, is important for the maintenance of our health. Recently, some studies have proposed new techniques that use sensors or cameras to measure or quantitatively evaluate vital signs [1,2,3,4,5,6,7] and, thus, facilitate their daily recording. However, the measurement of vascular function, which provides especially important data for disease prevention regarding circulatory conditions, is limited to clinical use because the operation of the associated measurement equipment is difficult for people other than professional medical staff. Therefore, to obtain vascular function data as readily as vital sign data, we require a new measurement system that anyone can easily operate.

Venous compliance (VC) has attracted attention because it provides important vascular function information. VC—i.e., the ability of a blood vessel to stretch—relates to various factors, such as blood vessel disease. Generally, decreased VC is associated with venous thromboembolism and studies show that VC is correlated with physical activity [8,9,10,11]. Moreover, it depends on age [12,13], and gender [14]. For these reasons, VC is an important indicator for health maintenance. 

The conventional method for measuring VC utilizes strain gauge plethysmography (SPG) [15,16], in which the volumetric change in a limb or digit, caused by a change in cuff pressure, is measured by a mercury-filled rubber strain gauge attached to the limb or digit. The change in blood volume (CBV) can be measured from this volumetric change. However, SPG suffers from a few crucial problems. First, movement of the limb or digit often causes errors in SPG measurements. Subjects are significantly inconvenienced because their bodies must be kept still for the duration of the measurement. Second, the contact area that can be measured by SPG is limited to parts around which the strain gauge can be wrapped, such as a limb or digit. In addition, the size of the strain gauge must be properly selected to fit the subject’s limb or digit, as inappropriate fitting of the gauge leads to measurement errors. Finally, SPG is affected by the damage of the strain gauge. 

To address these problems, we propose a non-contact plethysmography (NCPG) system that uses a red-green-blue (RGB) camera to measure VC [17]. We consider that there are two important differences between NCPG and SPG that may affect the resulting VC measurements. First, the blood vessels measured by the two systems are different. SPG measures all of the vessels within the strain gauge, whereas NCPG measures only the blood vessels located immediately under the skin because visible light cannot travel deeply into the skin tissue. Generally, deep blood vessels also need to be measured for the assessment of the venous system. Therefore, we must investigate whether there is a positive correlation between the VCs evaluated using the two systems. The NCPG method relies on diffuse reflected light, which reflects the absorption of light by the skin chromophore—i.e., the melanin, oxygenated hemoglobin, and deoxygenated hemoglobin. Second, NCPG is not affected by errors due to interstitial fluid accumulation, whereas such errors are possible in SPG measurements. When the upper arm is occluded with a pressure cuff, vein vasodilation is induced by the CBV due to arterial inflow. Even when arterial inflow reaches a plateau, the blood volume of the upper arm will continue to increase owing to the interstitial fluid, which can pass through the small holes of the capillary vessels and accumulate outside the blood vessels. Therefore, SPG measurements reflect not only the vasodilation but also the interstitial fluid accumulation. On the other hand, because hemoglobin does not pass through the small holes of the capillary vessels (see Section 2.5 for further details) NCPG can probe the vasodilation without errors due to interstitial fluid accumulation and, thus, provide a more correct estimation of VC with upper arm occlusion. Therefore, it is important to determine how these differences affect the CBV and VC measurements. In the present study, we compare CBV and VC results measured using the proposed NCPG approach with those obtained with the conventional SPG technique. 

Earlier studies [8,9,10,11] have shown a relationship between VC and physical activity. However, these reports were based on data that were measured using SPG. It is not clear whether NCPG would yield a similar result. Therefore, in this study, we categorized subjects into two groups and compared the mean VC of each group using the NCPG method.

Section 2 mainly reviews the core principles involved and the experimental setup of the dual measuring system. Specifically, these principles concern the estimation of blood concentration from the RGB values and the VC imaging method. Moreover, this section details two hypotheses regarding the two systems and the experimental conditions under which the measurements were performed. Section 3 presents and discusses the results. Finally, Section 4 provides brief concluding remarks.

## 2. Materials and Methods

### 2.1. NCPG for VC Using an RGB Camera Combined with SPG

**Figure 1:**
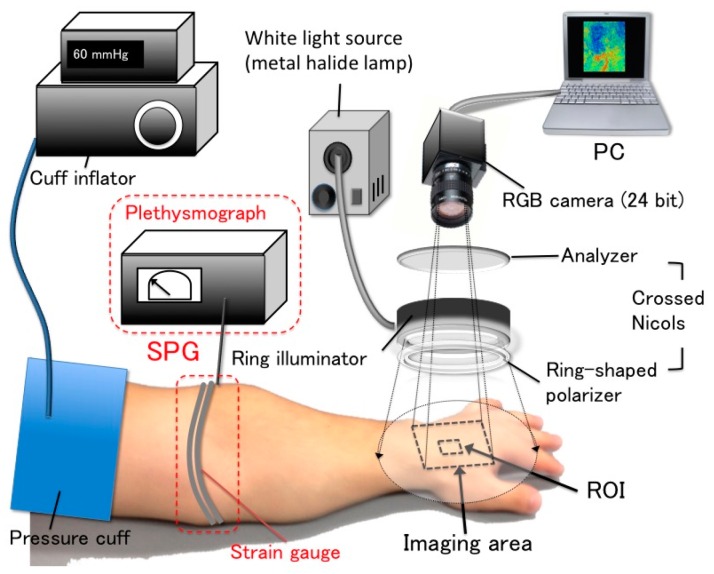
Setup of the simultaneous VC measurement system that consists of an NCPG and an SPG system.

Figure 1 shows the setup of the simultaneous measurement system that combined NCPG and SPG. The NCPG system consisted of a 24-bit RGB charge-coupled device (CCD) camera (DFK23U618, Imaging Source LLC, Charlotte, NC, USA) with a camera lens (Pentax/Cosmica, Japan; f16 mm, 1:1.4), a metal halide lamp (LA-180Me-R4, Hayashi, Tokyo, Japan), a ring illuminator, a set of polarization plates, a computer, a pressure cuff, and a cuff inflator (E-20, D. E. Hokanson Inc., Bellevue, WA, USA). The primary polarization plate (ring-shaped polarizer) and the secondary polarization plate (analyzer) were aligned in the crossed Nicols configuration to reduce specular reflection from the skin surface. The RGB camera was connected to the computer with a USB 3.0 cable. Before using the RGB camera on human skin, the inter-instrument differences in the output of the camera and the spatial non-uniformity of the illumination were corrected for using a standard white diffuser with 99% reflectance (SRS-99-020, Labsphere Inc., North Sutton, NH, USA). The SPG system consisted of a mercury-filled rubber strain gauge and a plethysmograph for data display (EC6, D. E. Hokanson Inc., Bellevue, WA, USA). The strain gauge carried an electric current at the time of measurement. Thus, when the strain gauge (which was wound around the limb) was stretched by the cuff inflation, the change in its electric resistance (which was measured by the plethysmograph) was proportional to the volumetric change in the limb. 

### 2.2. Estimation Method of Skin Chromophore Concentration from RGB Values

**Figure 2:**
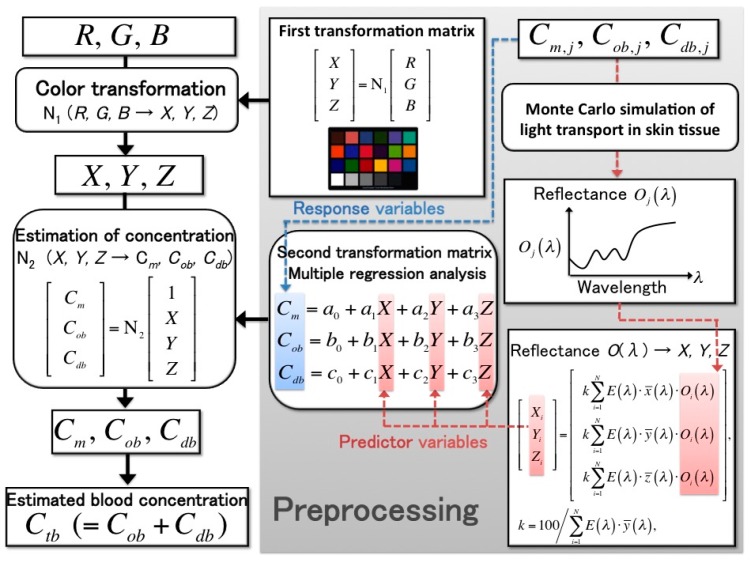
Procedure for estimating the melanin concentration *C*_m_, oxygenated blood concentration *C*_ob_, and deoxygenated blood concentration *C*_db_ from RGB values.

Any CBV in the skin tissue was estimated from the RGB images of human skin acquired using NCPG, whereas the blood volume was estimated from the volumetric change in a limb or digit using SPG. The method for estimating blood concentration from RGB values was proposed by Nishidate et al. [18,19,20]; this process is shown in Figure 2 and can be divided into three steps, as follows:

*Step 1*: We calculated the color transformation matrix **N_1_** that can transform RGB values into Commission Internationale de l’Éclairage XYZ (CIEXYZ) tristimulus values. The matrix **N**_1_ was determined in advance using a standard color chart. We measured the RGB values of a standard color chart (ColorChecker^®^, X-Rite Incorporated, Grand Rapids, MI, USA) consisting of 24 color chips. The diffuse reflectance spectra *O*(*λ*) for each chip were supplied with the chart. The CIEXYZ values for the specific illuminant *E*(*λ*) were calculated as follows:
  (1) $$\begin{array}{l} \left[\begin{array}{c} X_{i}\\ Y_{i}\\ Z_{i} \end{array}\right]=\left[\begin{array}{c} k\sum_{i=1}^{N}E\left(\lambda \right)\cdot \bar{x}\left(\lambda \right)\cdot O_{i}\left(\lambda \right)\\ k\sum_{i=1}^{N}E\left(\lambda \right)\cdot \bar{y}\left(\lambda \right)\cdot O_{i}\left(\lambda \right)\\ k\sum_{i=1}^{N}E\left(\lambda \right)\cdot \bar{z}\left(\lambda \right)\cdot O_{i}\left(\lambda \right) \end{array}\right]\\ k=100/\sum_{i=1}^{N}E\left(\lambda \right)\cdot \bar{y}\left(\lambda \right) \end{array}$$
  where *X_i_*, *Y_i_*, and *Z_i_* are the CIEXYZ values; $\bar{x}\left(\lambda \right)$, $\bar{y}\left(\lambda \right)$, and $\bar{z}\left(\lambda \right)$ are color-matching functions in the CIEXYZ color system; *N* is the total number of diffuse reflectance spectra; *k* is a constant whose value results in *Y* = 100 for a perfect diffuser. In this step, the XYZ value was calculated from each reflectance spectrum for 24 color chips (*N* = 24). Next, the transformation matrix **N**_1_ was determined using multiple regression analysis (MRA), in which the calculated XYZ values were the response variables and the measured RGB values were the predictor variables. The resulting regression coefficients were the elements of **N**_1_. If the RGB values of the color chips change by replacing the camera or the light source in the imaging system, the matrix **N**_1_ must be recalculated:
  (2) $$\left[\begin{array}{c} X\\ Y\\ Z \end{array}\right]=N_{1}\left[\begin{array}{c} R\\ G\\ B \end{array}\right]$$
*Step 2*: The second transformation matrix **N**_2_, which was used to transform the XYZ values into blood concentration values, was determined next. The skin chromophore concentrations—i.e., the melanin concentration *C*_m_, the oxygenated blood concentration *C*_ob_, and the deoxygenated blood concentration *C*_db_—represent the light absorption coefficients of the skin tissue. Using various combinations of these three concentrations, we employed a Monte Carlo simulation (MCS) model of the transit of light in human skin to calculate the diffuse reflectance spectra of the skin surface *O*(*λ*). The parameters of the MCS used in this study—such as the absorption coefficient, the scattering coefficient, the refractive index, and the layer thickness of skin tissue—were those employed in an earlier study [20]. Moreover, to determine matrix **N**_2_, we used the simulation to calculate 300 diffuse reflectance spectra in the wavelength range 400–700 nm at intervals of 10 nm [20]. After the XYZ values were calculated by integrating the diffuse reflectance spectra from Equation (1), which were obtained using MCS, the transformation matrix **N**_2_ was determined using MRA:
  (3) $$\left[\begin{array}{c} C_{m}\\ C_{\mathrm{ob}}\\ C_{\mathrm{db}} \end{array}\right]=N_{2}\left[\begin{array}{c} 1\\ X\\ Y\\ Z \end{array}\right]$$
*Step 3*: The XYZ values, which were transformed from the measured RGB values using the first matrix, **N**_1_, were transformed into the skin chromophore concentrations *C*_m_, *C*_ob_, and *C*_db_ using matrix **N**_2_. Thus, the total blood concentration was calculated as *C*_tb_ = *C*_ob_ + *C*_db_. The absorption coefficients of blood for the case of *C*_tb_ = 100% were set to those of blood with a 44% hematocrit and 150 g/L of hemoglobin.

### 2.3. Evaluation of VC

**Figure 3:**
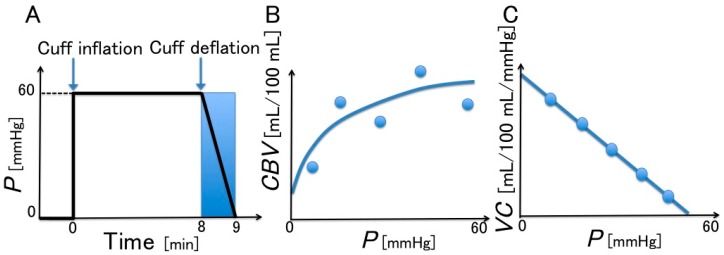
Method for the evaluation of VC (Halliwill et al. [21]). (**A**) Controlled cuff pressure; (**B**) change in blood volume (*CBV*) with respect to cuff pressure (*P*); and (**C**) venous compliance (*VC*) with respect to cuff pressure (*P*).

The method proposed by Halliwill et al. [21] for evaluating *VC*, shown in Figure 3, has been used in some recent studies. The procedure is as follows: as indicated in Figure 3A, after the cuff pressure (which is controlled by a cuff inflator) reaches 60 mmHg, it is maintained at that value for 8 min; it then decreases at a rate of 1 mmHg/s, which approaches the intravenous pressure [15], for 1 min while the volumetric change in the limb (around which the strain gauge is wrapped) is recorded using the plethysmograph. Figure 3B shows the regression equation for the volumetric change in the limb and Figure 3C displays the change in *VC* obtained by the differentiation of the regression equation. 

From the data obtained during the cuff deflation, the relationship between the CBV (mL/100 mL) and the cuff pressure (mmHg), which approximates the intravenous pressure, is obtained using the following quadratic regression equation:

(4) $$CBV=\;\beta_{0}+\beta_{1}P+\beta_{2}P^{2}$$

where the *CBV* obtained using SPG is Δ*BV*/*BV*_c_ (Δ*BV* = *BV* − *BV*_c_) and that using NCPG is Δ*C*_tb_/*C*_tb,c_, (Δ*C*_tb_ = *C*_tb_ − *C*_tb,c_); *BV*_c_ represents the blood volume and *C*_tb,c_ the blood concentration at the baseline (time = 0) and *BV* and *C*_tb_ denote these quantities at each measurement time; *β*_0_, *β*_1_, and *β*_2_ are constants; *P* is the cuff pressure. The *VC* (mL/100 mL/mmHg) at a particular cuff pressure is obtained from the differential of the regression equation:

*VC* = Δ*CBV*/Δ*P* = *β*_1_ + 2*β*_2_*P* (5)

where Δ*CBV* is the difference in the *CBV* and Δ*P* is the difference in the cuff pressure.

### 2.4. VC Imaging

**Figure 4:**
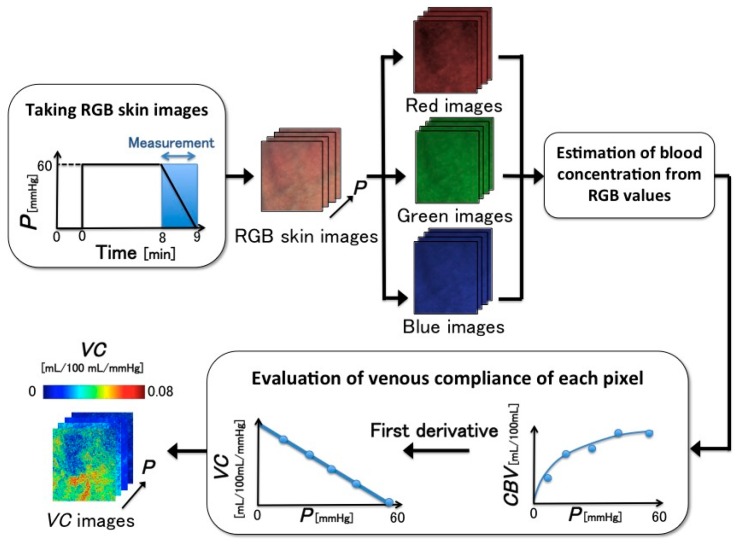
Proposed imaging process of *VC*, which is estimated from RGB images.

Figure 4 shows the proposed *VC* imaging process [17]. The spatial distribution of *VC* could not be observed because no visualization of *VC* was presented. Thus, we present a new *VC* imaging method that combines the evaluation of *VC* with an estimation of the blood concentration from the RGB values of each pixel. The new imaging method can easily obtain *VC* images from digital RGB images and provide *VC* measurements using a camera-equipped device, such as a smartphone. These images will help us understand *VC* intuitively. The new imaging process is as follows. First, during the 1-min period in which the pressure cuff is deflated from 60 mmHg to 0 mmHg, 60 RGB images of the skin are acquired. Secondly, as discussed in Section 2.2, the skin chromophore concentrations—i.e., *C*_m_, *C*_ob_, and *C*_db_—are estimated from the RGB value of each pixel. From this estimation, we can obtain the change of each pixel in terms of blood concentration. Thirdly, the *VC* of each pixel is determined by the change in the estimated blood concentration according to Equations (4) and (5). The *VC* images are obtained by calculating the *VC* of all pixels. This imaging process was programed using MATLAB R2015a (The Mathworks, Inc. Natick, MA, USA).

### 2.5. Differences between the Two Measurement Systems

In Section 1, we presented hypotheses concerning important differences that affect the measurement results of the NCPG and SPG methods. First, we considered that the measured blood vessels were different between the two methods and that this difference affects the results. The NCPG measurement is limited to superficial vessels, i.e., vessels near the skin surface. On the other hand, SPG can measure all vessels within the area covered by the strain gauge wound around the limb. 

Our second hypothesis is that the NCPG method is not affected by errors in interstitial fluid accumulation, whereas SPG is influenced by such errors. The walls of capillary vessels have small holes (called intercellular clefts) that supply nutrients to the cells. Moreover, fluid components exist inside and outside the blood vessels, namely, the blood plasma and interstitial fluid. Fluids can pass through the holes whereas proteins—such as hemoglobin—cannot. Four pressures, called Starling forces, determine the flow of the fluid [22]. Capillary pressure (*P_c_*) is the pressure in the blood vessel and is responsible for the fluid exiting the blood vessel. The interstitial fluid colloid osmotic pressure (Π*_i_*) also enables the flow of the fluid outside the blood vessel. On the other hand, the interstitial pressure (*P_i_*) and plasma colloid osmotic pressure (Π*_p_*) enable the flow of the fluid inside the blood vessel. Thus, assuming that inward pressure is positive and outward pressure is negative, the total pressure (*P_t_*) can be expressed as follows:

(6) $$P_{t}=P_{c}+\Pi_{i}-\left(P_{i}+\Pi_{p}\right)$$

The direction of the flow is determined mainly by the capillary pressure. If *P_c_* is higher than *P_i_* and Π*_p_*, the total pressure is positive (*P_t_* > 0) and the fluid flows outside the blood vessel. On the other hand, if *P_c_* is lower than *P_i_* and Π*_p_*, the total pressure is negative (*P_t_* < 0) and the fluid flows inside the blood vessel. The fluid flows inside the venous vessel because *P_c_* is lower and *P_t_* is negative. However, if the vein is occluded from the cuff pressure, the venous pressure becomes higher than usual. Thus, the fluid flows and accumulates outside the blood vessel. Since SPG measures the volumetric change in limbs, SPG measurements are affected by the fluid accumulation in the form of errors that have no relation to vasodilatation. To test these hypotheses, we conducted in vivo experiments using the two systems and compared the results.

### 2.6. Experimental Conditions

The experimental conditions of the NCPG measurements were as follows: As shown in Figure 1, the imaging area was the back of the subject’s left hand and the image size was 640 × 480 pixels. The lateral resolution of the resulting images was estimated to be 0.56 mm, which is insufficient to visualize the superficial veins. For example, the venula diameter is approximately 100–200 μm. Therefore, the *VC* images calculated using the method shown in Figure 4 are images in which the *VC* of superficial veins is spatially averaged. During the experiment, 660 frames of skin images were acquired at one frame per second (fps) by an RGB CCD camera, while 3300 measuring data were measured at five data points per second with the SPG method. Our region of interest (ROI) was 100 × 100 pixels and was placed on an area of the RGB skin image in which the vein could be observed.

The in vivo experiment was conducted on 11 male subjects (Japanese, mean age ± standard deviation: 24 ± 4 years) with no history or evidence of vascular diseases. 

In this study, we categorized the subjects into two groups—an active group and a sedentary group—according to their differences in physical activity. For each group, we compared the mean measured *VC* of the ROI and evaluated it using NCPG. For their categorization, we distributed a questionnaire to all subjects regarding their physical activity. The questionnaire only consisted of two questions (Q1 and Q2) as follows:

Q1 (Past habits): Did you perform any physical activity on a daily basis within the past year?
Q2 (Current habits): Do you perform any physical activity more than two hours per week?

If the subject answered yes to either question, he was categorized into the active group, whereas if he answered no to both questions, he was classified as sedentary. All experimental procedures were approved by Nippon Sport Science University Ethics Committee.

## 3. Results and Discussion

### 3.1. Images of Venous Compliances

**Figure 5:**
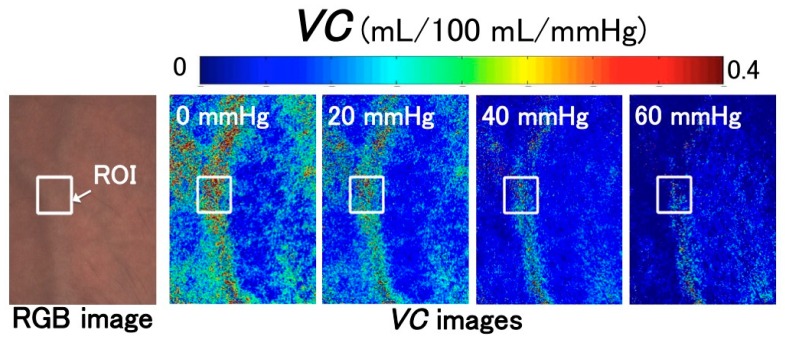
Acquired images showing the change in venous compliance (*VC*) depending on venous pressure. These images were obtained from the measurement of subject 3. ROI: region of interest. RGB: red-green-blue.

Figure 5 shows *VC* images constructed using the method shown in Figure 4. Figure 5 shows the RGB image of the skin and the change in the spatial distribution of *VC* with respect to the venous pressure, which corresponds to the change in cuff pressure during deflation. These images demonstrate that the area of greatest *VC* corresponds to the vein shown in the RGB image. Moreover, *VC* decreases with increasing venous pressure. This can be explained by the fact that the change in vein vasodilation decreases with increasing venous pressure. 

### 3.2. Difference between the Measurement Results of the Two Systems

**Figure 6:**
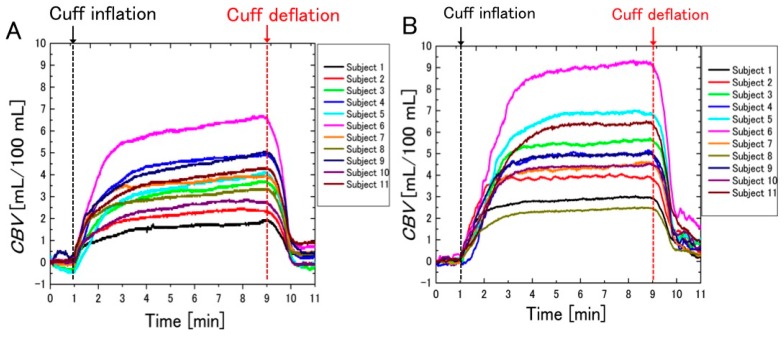
Temporal changes in blood volume (*CBV*s) measured with: (**A**) strain gauge plethysmography (SPG); and (**B**) non-contact plethysmography (NCPG) (*n* = 11).

Figure 6 shows the temporal change in blood volume under pressure, measured according to the method of Halliwill et al. using SPG and NCPG. The x-axis denotes time and the *y*-axis represents the *CBV*. Figure 6A shows the *CBV* that was calculated from the volumetric change in the strain gauge using SPG. Figure 6B shows the *CBV* that was calculated with the NCPG method from the color change of skin images—specifically, from the mean values of the ROI shown in Figure 5. 

**Figure 7:**
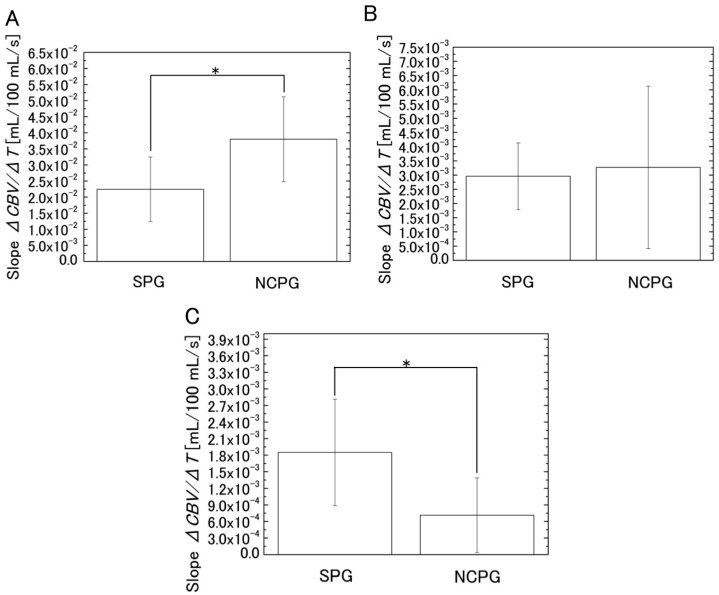
Estimated slope (mean ± SD) of the regression line calculated from the change in blood volume (*CBV*) measured with SPG and NCPG in three segments: (**A**) segment 1 (from 1 to 3 min); (**B**) segment 2 (from 3 to 6 min); and (**C**) segment 3 (from 6 to 9 min). * p < 0.05. SD: Standard deviation (*n* = 11).

**Figure 8:**
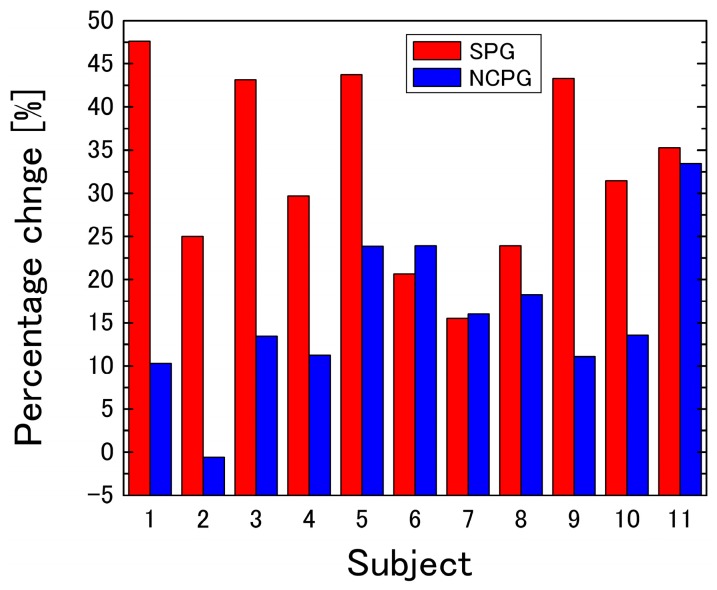
Percentage change in *CBV* between 3 and 9 min for each subject.

As shown in Figure 6, the *CBV* increases rapidly after cuff inflation. However, after a 1-min increase, the *CBV* measured with NCPG shows only a slight increase, whereas that obtained with SPG continues to increase until cuff deflation. To analyze the difference between the two methods, the slopes of the temporal change of the *CBV* were compared. For this analysis, the measurement time shown in Figure 6 was divided into three segments (segment 1 from 1 to 3 min, segment 2 from 3 to 6 min, and segment 3 from 6 to 9 min). The slopes (Δ*CBV*/ΔTime) of each segment were calculated from the regression lines of the temporal change of the *CBV* shown in Figure 6. In addition, after the normality of the mean values for each group was tested according to the Shapiro–Wilk test, the mean values of the slopes of the two systems were compared according to the Student’s *t*-test and the results are shown in Table 1. Figure 7 shows the estimated slope of the regression line calculated from the *CBV* measured with SPG and NCPG for each segment. As the results of the Shapiro–Wilk test show, all slopes had normality except for the slopes of NCPG at segment 2, which had no normality. As shown in Table 1, a significant difference was observed between two systems in segments 1 and 3. After the cuff was inflated, the *CBVs* of both systems increased significantly in segment 1. Then, all of the *CBVs* of SPG continue to gently increase in segments 2 and 3. On the other hand, as shown in Figure 6B and the standard deviation of NCPG in Figure 7B, some *CBVs* of NCPG increased significantly until 5 min in segment 2. Then, as shown in Figure 7C, the *CBV* increase of NCPG was less than that of SPG in segment 3. Moreover, Figure 8 shows the percentage change (%) in CBV between 3 and 9 min (=100 × (*CBV*_9 min_ − *CBV*_3 min_)/*CBV*_3 min_) for each subject, and Table 2 shows the results of a *t*-test comparing the percentage changes obtained with SPG and NCPG. As the table shows, a significant difference of the percentage change was observed between the two systems.

This discrepancy can be explained in terms of interstitial fluid accumulation. The rapid increase of venous blood pressure that results from cuff inflation leads to the fluid exiting the venous vessel. This flow leads to a volumetric change in the strain gauge that is not associated with vasodilatation, which leads to measurement errors. However, the NCPG method is not affected by such errors because it estimates the concentration of hemoglobin (which cannot exit the venous vessel) from the RGB values of the skin images. Therefore, these results provide strong evidence for the hypothesis presented in Section 1.

**Figure 9:**
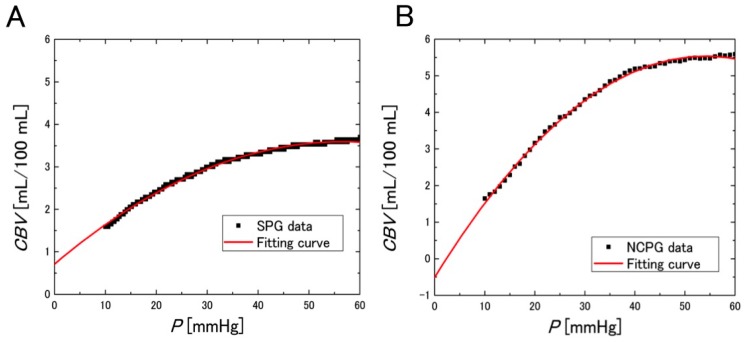
Observed *CBV* and quadratic regression curve with respect to the cuff pressure *P* during the deflation period, acquired with: (**A**) SPG (regression equation: *CBV* = 0.711 + 0.102·*P* − 8.99 × 10^−4^·*P^2^*, *R^2^* = 0.996, *p* < 0.0001); and (**B**) NCPG (regression equation: *CBV* = −0.503 + 0.223·*P* − 2.05 × 10^−3^·*P^2^*, *R^2^* = 0.998, *p* < 0.0001). The displayed data were obtained from the measurements of subject 3.

Figure 9 shows the *CBV* with respect to the cuff pressure during the deflation period and the quadratic regression curve. From the results, highly accurate regression equations were obtained. 

**Figure 10:**
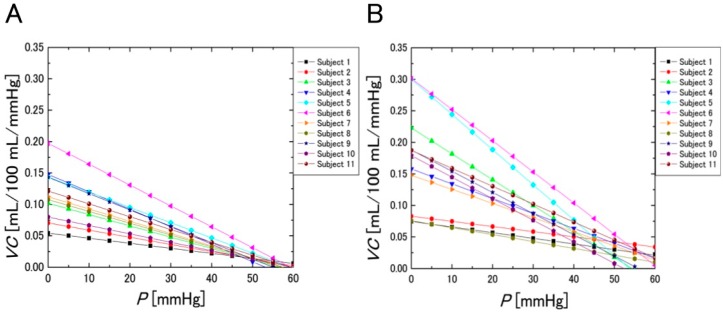
Change in *VC* evaluated by: (**A**) SPG; and (**B**) NCPG) (*n* = 11).

**Figure 11:**
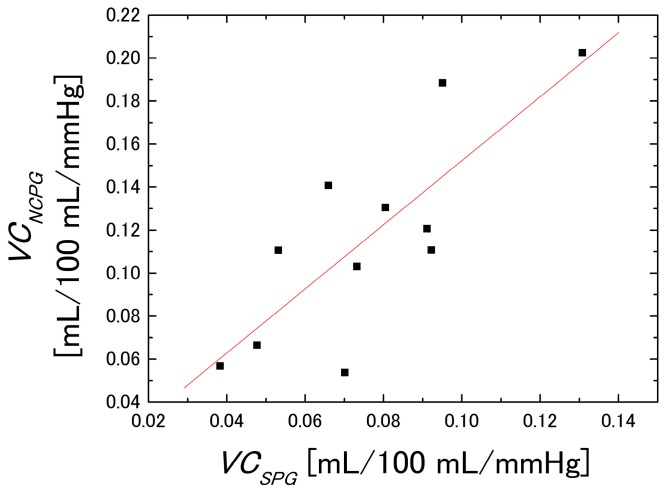
Correlation between *VC*_SPG_ (*VC* estimated using SPG) and *VC*_NCPG_ (*VC* estimated NCPG (*n* = 11).

**Figure 12:**
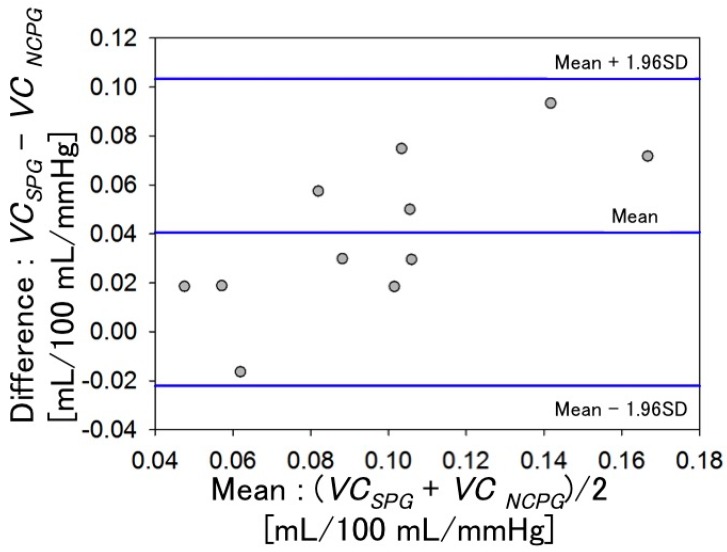
Bland-Altman comparison of *VC*_SPG_ and *VC*_NCPG_ (*n* = 11).

Figure 10 shows the *VC* evaluated using SPG and the NCPG. These figures were obtained by the primary differentiation of the regression equations with respect to the cuff pressure *P*. Figure 10A shows the *VC* obtained with SPG, whereas Figure 10B shows the mean *VC* evaluated within the ROI using the NCPG. To compare the *VC* values of the two graphs of Figure 10, we defined the *VC* value (mL/100 mL/mmHg) at 20 mmHg as the baseline and investigated the correlation between the *VC* values obtained using the two measurement systems, which is shown in Figure 11. The x-axis denotes the *VC* values of the subjects measured with SPG (*VC*_PSG_) and the y-axis those obtained with NCPG (*VC*_NCPG_). Pearson’s product-moment correlation coefficient between the two measurement systems is *R* = 0.797 (*p* < 0.005). Although differences exist between the two measurement systems, it can be concluded that there is positive correlation between the *VC*s evaluated using the two systems. Figure 12 shows the results of the Bland-Altman analysis for two *VC*s (*VC*_SPG_ and *VC*_NCPG_) measured using the two systems. The x-axis denotes the mean of the two *VC*s and the y-axis their difference. This result exhibits a difference between the two *VC*s. As mentioned previously, the difference indicates the difference in the vein types that were measured with the two systems (NCPG: superficial veins vs. (SPG: deep veins + superficial veins + interstitial fluids)). This also suggests a difference between the errors caused by interstitial fluid accumulation in the two systems.

### 3.3. Relationship between Physical Activity and VC

**Figure 13:**
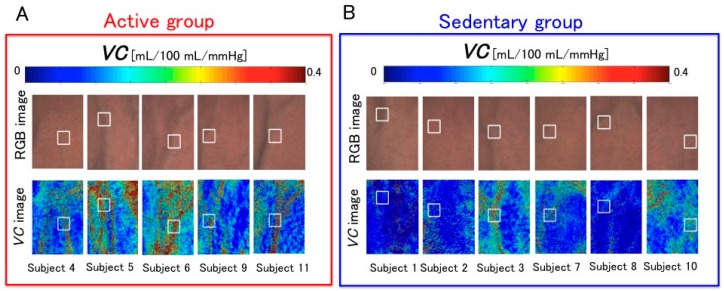
Resulting *VC* images obtained at 0 mmHg for subjects of: (**A**) The active group (*n* = 5); and (**B**) the sedentary group (*n* = 6).

Figure 13 shows the *VC* images of all subjects at 0 mmHg. In this study, all subjects were categorized into two groups—active and sedentary group—according to their differences in physical activity and the *VC* of each group was examined. By comparing the images of the two groups, we can observe that the *VC* values of the active group were relatively higher than those of the sedentary group. Thus, to statistically compare the *VC* values of the two groups, we defined the ROI and calculated the mean *VC* value over the ROI. Moreover, after the normality of the mean values for each group was tested according to the Shapiro-Wilk test, they were tested with the Student’s *t*-test. 

**Figure 14:**
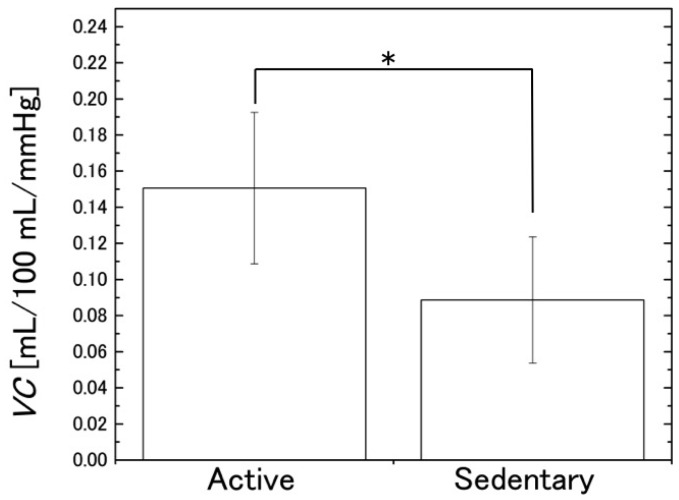
Comparison between the estimated *VC* values (mean ± SD) of the two subject groups (active and sedentary). * p < 0.05 (*n* = 11).

Figure 14 shows the comparison between the mean values of the two groups and Table 3 displays the results of the *t*-test. These results show that the mean *VC* in the active group was significantly higher than that in the sedentary group (*p* = 2.5 × 10^−2^). Similarly to the findings of earlier studies that used SPG [8,9,10,11], this indicates that *VC* evaluated with NCPG is associated with physical activity.

## 4. Conclusions

We achieved non-contact imaging by combining the evaluation of *VC* with a method that estimates the blood concentration from the RGB values of each pixel. Moreover, we simultaneously measured the *VC* of subjects using SPG and the proposed NCPG method and investigated the differences between these two systems. From the result shown in Figure 7, we can conclude that measurements performed with the proposed system are not affected by errors caused by interstitial fluid accumulation, whereas measurements with SPG are influenced by such errors. Moreover, we investigated the correlation between the *VC* values evaluated using the two systems. The results indicated a positive correlation (*R* = 0.797, *p* < 0.005) between *VC*_SPG_ and *VC*_NCPG_ and, thus, demonstrated that, in addition to SPG (i.e., the conventional *VC* measurement method), NCPG could be used for the evaluation of *VC*. Finally, we categorized subjects into two groups according to their differences in physical activity and compared the mean *VC* of each group using the non-contact system. The results showed that there was a significant difference between the mean *VC* of the two groups. Therefore, NCPG could investigate the relationship between *VC* and physical activity. 

In future studies, we will investigate the effectiveness of NCPG across a wider range of skin tones. Moreover, we will investigate the relationship between *VC* and various conditions, such as anemia, septic shock, hypovolemia, hypothermia, and obesity, among others.

## Figures and Tables

**Table 1:** Results of the *t*-test for the comparison between the slopes obtained with SPG and the NCPG.

Segment	System	Mean of Slope	SD of Slope	*t*-Value	*p*-Value
Segment 1	SPG	2.24 × 10^−2^	1.01 × 10^−2^	−3.11	5.47 × 10^−3^
	NCPG	3.80 × 10^−2^	1.32 × 10^−2^		
Segment 3	SPG	1.85 × 10^−3^	9.63 × 10^−4^	3.21	4.38 × 10^−3^
	NCPG	7.14 × 10^−4^	6.76 × 10^−4^		

**Table 2:** Results of the *t*-test for the comparison between the percentage changes obtained with SPG and NCPG.

System	Mean of Percentage Change	SD of Percentage Change	*t*-Value	*p*-Value
SPG	32.7	10.8	3.98	7.38 × 10^−4^
NCPG	15.9	8.95		

**Table 3:** Results of *t*-test for the *VC* of the active and sedentary groups.

Group	Number of Subjects	Mean of *VC* at 20 mmHg	SD of *VC* at 20 mmHg	*t*-Value	*p*-Value
Active group	5	0.151	0.042	2.68	0.025
Sedentary group	6	0.089	0.035		

## Abbreviations

VC	Venous compliance
SPG	Strain gauge plethysmography
NCPG	Non-contact plethysmography
RGB	Red-green-blue
CBV	Change in blood volume
CCD	Charge-coupled device
CIEXYZ	Commission Internationale de l’Éclairage XYZ
MRA	Multiple regression analysis
MCS	Monte Carlo simulation
ROI	Region of interest
